# Carbapenemase-Producing *Raoultella Planticola*: A Rare Cause of Pneumonia and Bacteremia

**DOI:** 10.3390/diseases6040094

**Published:** 2018-10-17

**Authors:** Jose Armando Gonzales Zamora, Monica Corzo-Pedroza, Maria Romero Alvarez, Octavio V. Martinez

**Affiliations:** 1 Division of Infectious Diseases, Department of Medicine, Miller School of Medicine, University of Miami, Miami, FL 33136, USA; monica.corzopedrosa@jhsmiami.org (M.C.-P.); maria.romeroalvarez@jhsmiami.org (M.R.A.); 2 Department of Pathology and Microbiology, Miller School of Medicine, University of Miami, Miami, FL 33136, USA; omartinez@med.miami.edu

**Keywords:** *Raoultella*, bacteremia, pneumonia, carbapenemase

## Abstract

*Raoultella planticola* is a gram-negative bacterium of the *Enterobacteriaceae* family that is usually found in soil, plant and aquatic environments. It is an uncommon human pathogen and has been associated with cases of bacteremia, pneumonia, urinary tract infections, among others. Here, we present the case of an 85-year-old female that developed nosocomial pneumonia and bacteremia caused by *Raoultella planticola*. Pertinent microbiological studies detected carbapenemase production codified by the bla_KPC_ gene. The patient was successfully treated with ceftazidime/avibactam and polymyxin. Our case illustrates the pathogenic potential of this organism and highlights the importance of phenotypic and genotypic assays for the appropriate identification of carbapenemase production.

**Figure 1:**
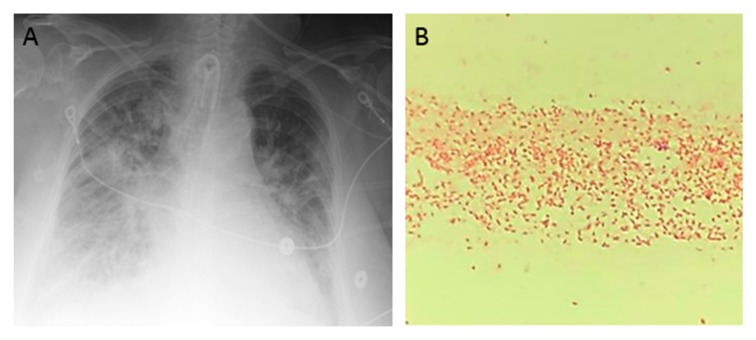

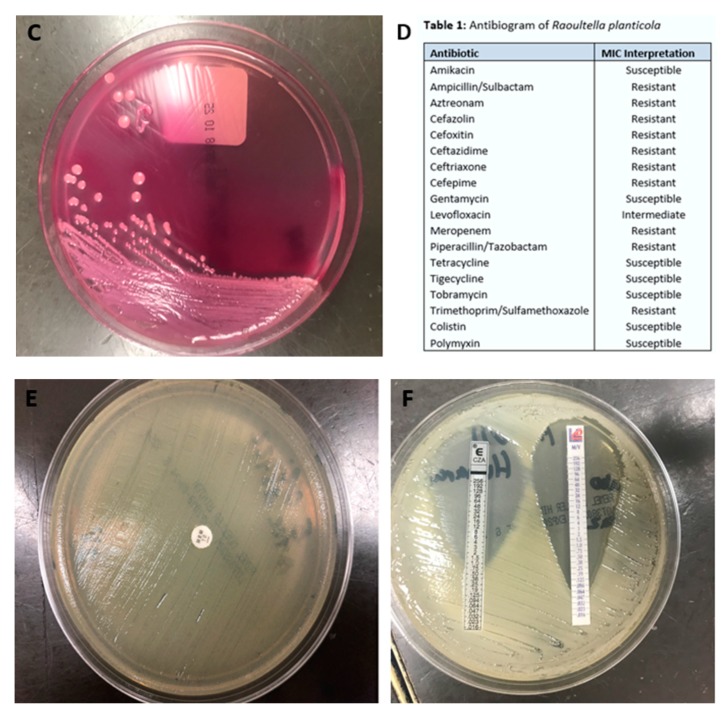
An 85-year-old woman presented to the hospital after sustaining burn injuries and smoke exposure from a house fire. On admission, she was tachypneic, with a respiratory rate of 20 breaths per minute. Her physical exam was significant for 2nd degree burns along the dorsal and palmar aspects of her first three fingers on the right hand and partially on the index finger and thumb of her left hand. She had 1st degree burns on the rest of her hands and both forearms, as well as on the entire face and neck. Her wounds were debrided and silvedene was applied on her hands and forearms, while bacitracin was used for her face. Shortly after admission, the patient experienced difficulty breathing, trouble swallowing, voice changes and swelling around her mouth and tongue. She was intubated for airway protection and was transferred to the intensive care unit (ICU). She was extubated 2 days later, but re-intubated after an episode of projectile vomiting and aspiration. Due to the development of fevers, blood and urine cultures were taken. She was started on cefepime 2 g every 12 h and vancomycin 15 mg/kg every 12 h for aspiration pneumonia. Blood culture grew methicillin-sensitive *Staphylococcus aureus* and urine culture was positive for *Morganella morganii.* Four days later, she developed spontaneous pneumothorax that required chest tube placement. Persistence of fever and worsening leukocytosis prompted antibiotic escalation to meropenem with continuation of vancomycin. Over the next 2 days, the patient developed acute renal failure and was started on continuous veno-venous hemodialysis. She completed 12 days of antibiotics. Over the next week, she developed a central line bloodstream infection caused by *Enterococcus faecalis* and ventilator associated pneumonia caused by methicillin-sensitive *Staphylococcus aureus*, for which she was treated with ampicillin/sulbactam for 7 days. Three days later, the patient developed a new episode of fever and worsening bilateral infiltrates in chest X-ray (**A**). Cultures were obtained, and gram stain from blood culture and endotracheal aspirate revealed gram-negative bacilli (**B**). The Mac Conckey agar demonstrated a pink color, which is characteristic of lactose-fermenting organisms (**C**). The patient was started empirically on cefepime 2 g every 12 h, and the central lines were removed (one of the potential sources of infection). The gram-negative rod was identified as *Raoultella planticola* by MALDI-TOF (matrix-assisted laser desorption ionization time of flight), and further in-vitro susceptibility testing by the Vitek^®^ 2 system (BioMérieux, Inc., Hazelwood, MO, USA) revealed resistance to most antibiotics, except for colistin, polymyxin B, aminoglycosides, tetracycline, and tigecycline (**D**). We performed additional phenotypic tests and detected carbapenemase production by the Carbapenem Inactivation Method (**E**). Supplementary antibiotic susceptibility studies by E-test showed susceptibility to ceftazidime/avibactam (**F**, left side) and meropenem/vaborbactam (**F**, right side). Confirmation of carbapenemase production was carried out by genotypic analysis using Verigene system (Luminex Corp, Austin, TX, USA), which identified bla_KPC_ and bla_CTX-M_ genes. Treatment with cefepime was discontinued and the patient was started on combination therapy with ceftazidime/avibactam and polymyxin B. Follow-up blood cultures were negative and her respiratory status improved over the following days. Finally, she was transferred to a LTAC (long-term acute care) facility to complete 2 weeks of antibiotic therapy.

*Raoultella planticola* is a non-motile, aerobic, encapsulated, gram-negative bacterium that belongs to the *Enterobacteriaceae* family [1]. It is usually found in soil, plant and aquatic environments. Cases of human infections by this organism has been described sporadically, mainly causing bacteremia, pneumonia, cholangitis, retroperitoneal abscess, among others [2]. The growing utilization of automated identification systems has allowed the identification of more infections caused by *Raoultella*, a pathogen once classified as *Klebsiella* given its biochemical similarities [2]. The majority of the patients affected by this pathogen have certain degree of immunosuppression (malignancies, recent chemotherapy or post-transplant) or have a history of prolonged ICU hospitalization or recent trauma. In the present case, our patient developed pneumonia and bacteremia secondary to *Raoultella* after 40 days of ICU stay. Initial reports have described *Raoultella* as a low virulence pathogen, in which treatment with cephalosporins and carbapenems usually leads to excellent outcomes [1]. However, this perception has changed in recent years with the more frequent isolation of multidrug-resistant organisms with the capability of carbapenemase production. In a recent review, Xu et al. described the emergence of strains of *Raoultella planticola* with carbapenemase-producing genes, such as bla_KPC_, bla_IMP_, and bla_NDM_ [3]. A total of six cases were reported in this review that included patients with pneumonia and bacteremia, whom prognosis was mostly fatal (4/6 deaths). Since then, four more cases of carbapenemase-producing *Raoultella planticola* have been reported in the literature, three of them carrying the bla_OXA_ gene and one isolate with bla_NDM_ [2,4]. The current availability of appropriate microbiological studies for the identification of carbapenemase production has facilitated the evaluation and treatment of patients that carry these isolates. Many phenotypic assays have been developed, of which the Carbapenem Inactivation Method is recognized as one of the most accurate, with a sensitivity of 91% and a specificity of 99% [5]. A huge progress has also taken place in genotypic assays, with several commercial methods that allow precise identification of carbapenemase producing genes. In our case, we used the Verigene system (Luminex Corp, Austin, TX, USA), that detects the five most common genes that codify carbapenemases. The treatment of these infections is very challenging, with more studies favoring combination therapy [6]. We opted for the use of ceftazidime/avibactam and polymyxin, obtaining very good outcomes. Our case illustrates the pathogenic potential of *Raoultella planticola* and highlights the importance of phenotypic and genotypic tests for the appropriate identification of carbapenem-resistant strains.
